# Supplementary material for: Zonulin-Dependent Intestinal Permeability in Children Diagnosed with Mental Disorders: A Systematic Review and Meta-Analysis
Source: Nutrients. 2020 Jul 3;12(7):1982. doi: 10.3390/nu12071982 (PMC7399941; doi:10.3390/nu12071982)
Supplement: Supplementary file 1 [file nutrients-12-01982-s001.pdf]

Table S1. Summary of population characteristics.

References	Eligibility criteria	Age, years	Male/female proportion	Prior treatment history
Özyurt et al., 2018	Patients: Recruited at the Child Psychiatry and Pediatrics outpatient clinic of Tepecik Research and Training Hospital in Turkey. Chronological age of 7-12 years diagnosed with ADHD according to DSM-5 criteria and providing verbal assent (for children) and written informed consent (for parents). Controls: Recruited at the pe- diatric well-child clinics of the study center<br>Chronological age of 7-12 years, no psychopathology (K-SADS-PL), no chronic diseases/allergies/immunological disorders requiring treatment, providing verbal assent (for children) and written informed consent (for parents) for participation.	7-12	51/30	N/A
Esnafoglu et al., 2017	Patients: From Ordu University Faculty of Medicine Education and Research Hospital in Turkey - Pediatrics and Adolescent Psychiatry outpatient clinics with ASD diagnosis according to DSM-5 criteria. Controls: were included from those applying to the pediatric and adolescent psychiatry and pediatric outpatient clinics for minor reasons at the same hospital.	4-11	46/19	N/A
Józefczuk et al., 2017	Patients: with ASDs. ASDs based on diagnostic interviews, observations, and examinations performed by pediatricians, neurologists, and psychologists. Patients did not attach GCFD for at least six months. Controls: Sera from healthy controls.	2.5-18	N/A	N/A
Rose et al., 2018	Enrolment in the Childhood Autism Risk from Genetics and Environment (CHARGE) study (Hertz-Picciotto et al. 2006). Participants were recruited from 4 groups, 1) children with ASD and GI symptoms of irregular bowel movements, 2) children with ASD, and no GI symptoms 3) typically developing children with GI symptoms of irregular bowel movements 4) typically developing children without GI symptoms.	3-12	63/24	N/A
Işık et al., 2020	Patients from the outpatient clinic for child and adolescent psychiatry at the Suleyman Demirel University Medical Faculty in Turkey.) All patients were screened for psychiatric disorders according to The Schedule for Affective Disorders and Schizophrenia for School-Aged Children, Present and Lifetime Version (K-SADS-PL. Exclusion criteria were implemented: the existence of psychiatric disorders such as mental retardation, ASD, schizophrenia, bipolar disorder, and major depression; major physical (such as diabetes mellitus), allergic, or neurological diseases; those with body mass index (BMI) percentile ≥95%; use of corticosteroids or a drug that affects the immunological system in the last 6months; having an active infection within the past month; and clinical suspicion of PANDAS or PANS. Controls: Healthy subjects matched by age and gender who applied to the hospital for a routine check-up. The same exclusion criteria were performed for the control group that was not affected by a major mental disorder.	10-18	26/22	Sixteen patients were undergoing treatment. Ten patients used selective serotonin reuptake inhibitors (SSRI), and six took an SSRI and antipsychotic combination therapy.

ADHD: Attention deficit hyperactivity disorder K-SADS-PL: Kiddie Schedule for Affective Disorders and Schizophrenia. ASD: Autism spectrum disorder. DSM-5: Diagnostic and Statistical Manual of Mental Disorders, 5th edition. GCFD: gluten-/casein-free diet. GI: Gastro-intestinal. SSRI: Selective serotonin reuptake inhibitors. BMI: Body mass index.

Table S2. Participants characteristics.

References	Age, years.	Gender	Disease characteristics	Diagnostic criteria
Özyurt et al., 2018	7-12	Male and female	Developmentally inappropriate and impairing symptoms of inattention, hyperactivity, and impulsivity. Problems with social cues, misinterpretation of social situations, and inappropriate responding.	DSM-5, DuPaul ADHD scale, and the Social Responsiveness Scale (SRS).
Esnafoglu et al., 2017	4-11	Male and female	High rate of gastro-intestinal (GI) symptoms like diarrhea, constipation, pickiness about food, bloating, and stomach pain.	DSM-5
Józefczuk et al., 2017	2.5-18	Male and female	Impaired social interaction, communication, and repetitive behavior were appearing before three years of age.	ASD diagnostic criteria based on diagnostic interviews, observations, and examinations performed by pediatricians, neurologists, and psychologists.
Rose et al., 2018	3-12	Male and female	Social impairment and presence of repetitive or stereotyped behaviors.	DSM-5, Autism Diagnostic Interview-Revised (ADI-R) and Autism Diagnostic Observation Schedule (ADOS). Participants in the typically developing group had to score within the typical range on Social Communication Questionnaire (SCQ), Mullen Scales of Early Learning (MSEL), and Vineland Adaptive Behavior Score (VABS).
Işık et al., 2020	10-18	Male and female	Distinctive and recurrent obsessions and/or compulsions.	DSM-5 criteria for OCD

Table S3. Summary of metabolic parameters.

References	Biomarkers
Özyurt et al., 2018	Serum zonulin levels.
Esnafoglu et al., 2017	Serum zonulin levels.
Józefczuk et al., 2017	Celiac specific antibodies (against intestinal TG2, neural TG6, and DPG) and AGA, in both IgA and IgG classes, I-FABP, zonulin.
Rose et al., 2018	Cytokine analyses (IL-1α, IL-1β, IL-6, IL-12(p40 & p70)), (TNF)α, IFNγ (TH1), IL-4, IL-13(TH2), IL-10, (TGF)β1, IL-5, IL-15, IL-17, haptoglobin plasma levels, total DNA from stool samples.
Işık et al., 2020	Serum zonulin and claudin-5 levels.

Table S4. Plasma haptoglobin levels

Groups	*n*	Haptoglobin genotypes					
		Hp1-1		Hp2-1		Hp2-2	
		n	Plasma levels	*n*	Plasma levels	*n*	Plasma levels
Total ASD (*n*)	54	5	9.3	29	53.7	20	37
ASD GI (*n*)	23	2	8.7	12	52.2	9	39.1
ASD NoGI (*n*)	31	3	9.7	17	54.8	11	35.5
Total TD (*n*)	43	8	18.6	19	44.2	16	37.2
TD GI (*n*)	6	2	33.3	3	50	1	16.7
TD NoGI (*n*)	37	6	16.2	16	43.2	15	40.5

ASD: autism spectrum disorders, ASD - GI: with ASD and GI symptoms of irregular bowel habits, ASD - NoGI: with ASD but without current or previous GI symptoms, TD-GI: typically developing children with GI symptoms, TD-NoGI: typically developing children but without current or previous GI symptoms.
